# P35 Pyrexia of unknown origin in a patient with long standing rheumatoid arthritis

**DOI:** 10.1093/rap/rkae117.066

**Published:** 2024-11-05

**Authors:** Syed Parves, David Garner, Kiki Mistry, Helen Powell, Arun Sahai, Sangita Agarwal, Mark Lloyd

**Affiliations:** Frimley Park Hospital, Frimley, United Kingdom; Frimley Park Hospital, Frimley, United Kingdom; Frimley Park Hospital, Frimley, United Kingdom; Frimley Park Hospital, Frimley, United Kingdom; Guy’s and St Thomas’ Hospital, London, United Kingdom; Guy’s and St Thomas’ Hospital, London, United Kingdom; Frimley Park Hospital, Frimley, United Kingdom

## Abstract

**Introduction:**

Pyrexia of unknown origin (PUO) can be challenging in treated rheumatic auto-immune disease. Many conditions may have fever as part of the symptom complex, treatment may mask signs and symptoms and can also make opportunistic infection more likely. Unusual or fastidious infections can be particularly difficult to diagnose. We describe a case of ureaplasma infection that evaded detection for a prolonged period in a female with long standing rheumatoid arthritis (RA).

**Case description:**

47 year old female presented January 2023 with fevers, weight loss and persistently high CRP. RA diagnosed in 1998. Erosive wrist and MTP disease, initial RF 32, CCP 8. Treatment- 5-10 mg prednisolone since 1998, adalimumab 2010, rituximab 2013-2022. Normal Ig levels, IgG lamda paraprotein since 2016 (too low to quantify). Frequency and dysuria March 2022. Cystoscopy- small bladder; biopsies in May 2022 and August 2023 showed acute on chronic inflammatory infiltrate with granulation tissue. Interstitial cystitis diagnosed. She had recurrent episodes of pyuria and was given several courses of antibiotics, including meropenem, temocillin, fluconazole, gentamicin. Despite the recurrent pyuria no organisms were cultured.

CT CAP February 2023 -ground glass opacity right lower lobe. Later CXR clear. Quantiferon negative, also negative 2016, 2019. Other normal / negative tests- TTG, HIV, Hep ABCE, TFTs, EBV viral load.

EBV, CMV IgG +ve. CMV titres 696 c/ml June 2023. CD 19 undetectable. IgG4 0.02 g/L. MPO, PR3 negative. BK virus was detected in urine. There was no sustained response to iv methylprednisolone and a course of filgotinib given for possible systemic RA symptoms.

She developed a dry cough and shortness of breath. PET-CT July 2023 - bilateral FDG avid large confluent areas of consolidation. Urine + bronchial washings were negative for TB growth at 42 days. However, in view of deterioration empirical TB treatment was started July 2023.

CT urogram in September 2023 showed likely bilateral renal abcesses, as well as bilateral hydronephrosis. Left renal cyst aspirate was performed and empirical doxycycline started at 100 mg bd. Ureaplasma detected from the aspirate on PCR. 3 weeks doxycyline given with rapid resolution of fevers and CRP. February 2024- cystectomy and ileal conduit in for refractory interstitial cystitis with a poorly compliant, small capacity, thick walled fibrotic bladder which had caused bilateral ureteric obstruction. She has made a good recovery. TB treatment course was completed.

**Discussion:**

This case shows the difficulties in diagnosing infection from a little known and difficult to detect organism, ureaplasma. Discovered in the 1950s, it has no cell wall and very small genome and is therefore an obligate parasite /saprophyte. Together with mycoplasma it belongs to the bacterial class of Mollicutes. It cannot be routinely cultured nor gram stained. PCR or specialist culture is required. It may be present as a commensal but has been implicated in symptomatic genitourinary infections. It has also been implicated in native and non-native joint infections, and in infants can cause a pneumonic illness. Empirical treatment may be indicated due to long turn around times for diagnosis. There is some evidence that reduced humoral immunity may be a contributory factor to infection. We have found a previous case described of ureaplasma renal abcesses in a patient with MS and neurogenic bladder treated with rituximab. Our patient had normal immunoglobulin levels though still had profound CD19 suppression. Further immune suppression resulted from methylprednisolone and course of filgotinib, which may have accelerated the course of infection. Ureaplasma and mycoplasma do not have a cell wall, thus making beta-lactam antibiotics and vancomycin ineffective. Cyclines, josamycin and fluroquinolones are effective, though there is increasing resistance developing. We gave a relatively long course of doxycycline as our patient was very unwell and had apparent extensive urinary tract involvement. BK virus seropositivity is present in 90% of the normal population and asymptomatic viruria in up to 20%. However, it is a cause of renal tract inflammation in the immunosuppressed – in particular post-transplant. We felt it was unlikely to be significant in our patient in view of the clinical picture, lack of BK viraemia and negative viral findings on bladder biopsy.

**Key learning points:**

• Ureaplasma should be on the list of organisms to consider in cases of PUO when the more common infective causes have been ruled out, particularly in immune suppressed patients. Patience and close collaboration with colleagues is essential. This patient had multiple MDT meetings involving infectious disease, respiratory, secondary and tertiary care urology and rheumatology specialists. The collaboration was invaluable. A course of doxycycline may be considered as a relatively low risk approach while awaiting microbiological confirmation in such cases.

